# Evaluation of the In Vitro Activity of Bedaquiline, Delamanid, and Clofazimine Against *Mycobacterium abscessus* Complex and Their Antibiofilm Potential

**DOI:** 10.3390/pathogens14060582

**Published:** 2025-06-11

**Authors:** Katarzyna Kania, Katarzyna Wójcik, Alicja Skórkowska, Karolina Klesiewicz

**Affiliations:** 1 Department of Pharmaceutical Microbiology, Faculty of Pharmacy, Jagiellonian University Medical College, St. Medyczna 9, 30-688 Krakow, Poland; ka.kania@uj.edu.pl; 2 Laboratory of Microbiology, The St. John Paul II Specialist Hospital, St. Pradnicka 80, 31-202 Krakow, Poland; 3 Malopolska Central Laboratory of Tuberculosis Diagnostics, The St. John Paul II Specialist Hospital, St. Ulanow 29, 31-455 Krakow, Poland; k.wojcik@szpitaljp2.krakow.pl; 4 BioImaging Laboratory, Center for the Development of Therapies for Civilization and Age-Related Diseases, Jagiellonian University Medical College, Medyczna 9, 30-688 Krakow, Poland; alicja.skorkowska@uj.edu.pl

**Keywords:** *Mycobacterium abscessus* complex, non-tuberculous mycobacteria, bedaquiline, biofilm formation

## Abstract

*Mycobacterium abscessus* complex (MABc) poses a major therapeutic challenge due to its intrinsic multidrug resistance and ability to form biofilms. This study evaluated the in vitro activity of three antimycobacterial agents—bedaquiline, delamanid, and clofazimine—on 20 clinical MABc isolates, including *M. abscessus* subsp. *abscessus*, *massiliense*, and *bolletii*, with a focus on biofilm-forming phenotypes. Biofilm analysis showed that the rough colony morphotypes were mostly weak biofilm formers, while the smooth and mixed morphotypes were predominantly moderate or strong biofilm formers. A statistically significant association was observed between the mixed colony morphology and strong biofilm formation (*p* = 0.032). Importantly, bedaquiline exhibited potent and consistent activity across all isolates, regardless of the biofilm-forming ability, with MIC values ranging from 0.125 to 1 µg/mL. In contrast, delamanid and clofazimine showed limited efficacy, with MIC values exceeding 16 µg/mL and 8 µg/mL, respectively. These findings strongly support the role of bedaquiline as a promising core agent for future combination therapies targeting drug-resistant MABc infections, including biofilm-associated infections. Our results, among the first from Poland, highlight the critical need for incorporating novel agents such as bedaquiline into therapeutic strategies against this difficult-to-treat pathogen.

## 1. Introduction

*Mycobacterium abscessus* complex (MABc) is a rapidly growing non-tuberculous mycobacteria (NTM) that has emerged as a significant pathogen, causing severe pulmonary and extrapulmonary infections [1,2]. Treating patients with underlying conditions such as cystic fibrosis, chronic obstructive pulmonary disease, or those undergoing immunosuppressive therapy is particularly challenging [3,4,5,6]. In addition, the treatment of MABc is complicated by its intrinsic resistance to many antimicrobial agents and the ability of mycobacteria to form biofilms [7,8,9]. Resistance mechanisms include changes in genes encoding the drug’s molecular targets, inherent resistance or tolerance, and the limited permeability of the cell wall to drug penetration [10,11,12]. Biofilm formation increases antibiotic resistance, and protects bacteria from the host’s immune defences, making infections harder to treat [13,14,15].

The management of MABc infections remains a significant clinical challenge due to intrinsic resistance mechanisms and limited therapeutic options, highlighting the critical importance of performing susceptibility testing using a standardised antimicrobial panel to guide effective treatment. *M. abscessus* is particularly challenging to treat, with success rates ranging between 41% and 46% for all subspecies [16]. The recommended agents include amikacin, cefoxitin, ciprofloxacin, clarithromycin, doxycycline (or minocycline), imipenem, linezolid, moxifloxacin, trimethoprim-sulfamethoxazole, and tobramycin [17]. For *M. abscessus* subsp. *abscessus* isolates, additional testing for tigecycline and clofazimine may be warranted [17]. However, due to insufficient clinical correlation with in vitro data, the susceptibility breakpoints for these agents remain undefined, necessitating the reporting of minimum inhibitory concentrations (MICs) instead [17,18,19]. The effective use of antibiotics such as imipenem and clarithromycin is highly dependent on precise species-level identification, which should be performed using molecular methods [17].

Among the emerging therapeutic options, bedaquiline, a diarylquinoline, has shown promise for *M. abscessus* treatment [20]. However, there is limited information regarding its use for treating *M. abscessus* infections. By targeting ATP synthase, it disrupts bacterial energy metabolism and exhibits bactericidal activity. Although primarily approved for multidrug-resistant *Mycobacterium tuberculosis* (MDR-TB) [21,22], studies indicate that bedaquiline may have significant activity against *M. abscessus*, including drug-resistant and biofilm-forming strains [23,24,25].

Similarly, delamanid, a nitroimidazo-oxazole derivative, is another promising agent in the fight against *M. abscessus* [25,26]. It inhibits mycolic acid synthesis, a key component of the mycobacterial cell wall, effectively disrupting bacterial growth [27,28]. While delamanid has been approved for MDR-TB treatment, its potential against NTMs like MABc is an area of emerging research, with preliminary findings suggesting its efficacy in combination therapies [25]. Clofazimine, a lipophilic antibiotic, was initially developed for the treatment of leprosy [29,30,31]. In recent years, it has regained attention for its potential role in treating MDR-TB [32,33,34] and has also been discussed in the management of *M. abscessus* infections [35,36,37]. Its dual bactericidal and anti-inflammatory properties make it particularly effective against persistent bacterial populations, including those within biofilms. Clofazimine’s synergy with other drugs, such as bedaquiline, further enhances its therapeutic potential, especially in multidrug-resistant cases [37].

Biofilm formation remains a critical factor in MABc pathogenicity, contributing to its persistence and resistance [38]. Biofilms are complex bacterial communities encased in a self-produced extracellular matrix that provides protection from environmental stresses and antimicrobial agents. Understanding the interplay between biofilm formation and drug resistance is essential for developing effective therapeutic strategies [39].

This study explored the activity of bedaquiline, delamanid, and clofazimine against *M. abscessus* complex strains, with a focus on biofilm-forming isolates. Furthermore, we evaluated the MABc strains and correlated their phenotypes with drug susceptibility, providing insights into more effective diagnostic and therapeutic approaches.

## 2. Materials and Methods

### 2.1. Mycobacterial Strains

The study analysed 20 clinical strains of the *M. abscessus* complex isolated from pulmonary samples at the Central Laboratory of Tuberculosis Diagnostics, Saint John Paul II Specialist Hospital in Krakow, Poland, between January 2021 and September 2024. All isolates were identified as the causative agents of mycobacteriosis in accordance with the Infectious Diseases Society of America guidelines [17]. The strains were stored in cryobanks at −80 °C. After thawing, the strains were cultured on Löwenstein–Jensen (LJ) medium and, simultaneously, in liquid medium.

For Löwenstein–Jensen (LJ) culture, samples were incubated for 5–7 days to obtain visible colony growth. For the BACTEC MGIT 960 system, a full loop of bacterial growth from LJ medium was suspended in sterile saline solution. A 500 μL aliquot of the suspension was inoculated into BD BACTEC™ MGIT™ mycobacterial growth indicator tubes (BBL) (BD Diagnostics, Franklin Lakes, NJ, USA) containing liquid medium, supplemented with MGIT broth and PANTA™ (polymyxin B, amphotericin B, nalidixic acid, trimethoprim, azlocillin) and incubated at 37 °C in the BD BACTEC™ MGIT™ 960 instrument (BACTEC MGIT) (BD Diagnostics, Franklin Lakes, NJ, USA), until flagged positive by the system, typically after 5–7 days.

### 2.2. Molecular Identification of Subspecies Within the M. abscesuss Complex and Molecular Mechanisms of Resistance Assessed by the DNA Strip Method

The GenoType *Mycobacterium* assay was performed using total DNA extracted with the GenoLyse version 2.0 kit (Hain Lifescience, Bruker, Nehren, Germany) from monocultures of mycobacteria cultivated in liquid medium (BBL), within the BACTEC MGIT 960 system (BD Diagnostics, Franklin Lakes, NJ, USA), following the manufacturer’s protocol. The identification of the isolates as members of the MABc was confirmed using the CM Direct version 1.0 assay (Hain Lifescience, Bruker, Nehren, Germany) in accordance with the manufacturer’s protocol [40,41]. DNA identification was carried out via a hybridisation process coupled with an alkaline phosphatase reaction on a membrane strip. The amplification reagents, including polymerase and primers, were provided in mixes AM-A and AM-B. The amplification protocol comprised initial denaturation at 95 °C for 15 min, followed by 20 cycles of denaturation at 95 °C for 30 s and annealing at 65 °C for 120 s. This was followed by 30 cycles of denaturation at 95 °C for 25 s, annealing at 50 °C for 40 s, and extension at 70 °C for 30 s, concluding with a final elongation phase at 70 °C for 8 min. Molecular differentiation within the *M. abscessus* complex was performedusing the GenoType NTM-DR ver. 1.0 test (Hain Lifescience, Bruker, Nehren, Germany), following the manufacturer’s PCR protocol with specified primers and thermal cycling conditions. The 50 μL master mix included 10 μL of AM-A, 35 μL of AM-B reagents, and 5 μL of extracted DNA. The PCR cycle began with denaturation at 95 °C for 15 min, followed by 10 cycles (95 °C for 30 s, 65 °C for 120 s) and 20 cycles (95 °C for 25 s, 50 °C for 40 s, 70 °C for 40 s), ending with a final extension at 70 °C for 8 min. Hybridisation was performed using a manual protocol with a water bath or TwinCubator. After amplification, the samples were denatured with DEN solution, hybridised with prewarmed HYB buffer, and incubated at 45 °C for 30 min. The hybridised samples were then stringently washed with STR solution, and the strips were conjugated with diluted CON-C and incubated, followed by washing with RIN and distilled water. The substrate reaction with diluted SUB-C was conducted in the dark for 3–20 min until bands were visible. The strips were dried and interpreted according to the manufacturer’s guidelines [41].

### 2.3. Morphology of the Colony of Mycobacterial Strains

To distinguish the morphologies of *M. abscessus*, bacterial strains were inoculated onto Middlebrook 7H10 agar plates (BD Diagnostics, Franklin Lakes, NJ, USA) and incubated at 28 °C under aerobic conditions for up to 10 days. Colony growth was monitored daily. The morphology of colonies was evaluated directly on the agar plates using a light microscope (Zeiss Axiolab, equipped with ZEISS Axiocam 208 color, Carl Zeiss Microscopy GmbH, Jena, Germany) and visually. Smooth (S) morphotypes were identified by their moist, glossy, and translucent appearance with a uniform texture and rough (R) morphotypes were characterised by rugged, waxy colonies with pronounced cording patterns.

### 2.4. Phenotypic Drug Susceptibility Testing by Routinely Used Method

Drug susceptibility testing (DST) of NTM was carried out using the RAPMYCO Sensititre system (Thermo Fisher, Cleveland, OH, USA). Minimum inhibitory concentrations (MIC) were determined through microdilution in Sensititre Mueller–Hinton Broth (MHB) with TES (T3462), using 96-well polystyrene plates containing lyophilized antibiotics at a two-fold dilution. The plate contained antibiotics at doubling concentrations of the following drugs: amikacin (1–64 µg/mL), cefoxitin (4–128 µg/mL), ciprofloxacin (0.12–4 µg/mL), clarithromycin (0.06–16 µg/mL), doxycycline (0.12–16 µg/mL), imipenem (2–64 µg/mL), linezolid (1–32 µg/mL), minocycline (1–8 µg/mL), moxifloxacin (0.25–8 µg/mL), tigecycline (0.015–4 µg/mL), trimethoprim/sulfamethoxazole (0.25/4.75–8/152 µg/mL), cefepime (1–32 µg/mL), and amoxicillin/clavulanic acid in a 2:1 ratio (2/1–64/32 µg/mL), ceftriaxone (4–64 µg/mL), and tobramycin (1–16 µg/mL). MICs were recorded after 3–5 days of incubation if control growth was observed, except for clarithromycin, for which incubation was extended to 14 days to detect inducible macrolide resistance. The DST procedure was performed according to the manufacturer’s recommendations. In brief, 3–5 colonies were transferred into water to prepare a suspension with a 0.5 McFarland turbidity standard, as measured by a Nephelometer. A total of 50 μL of this suspension was combined with Sensititre Mueller–Hinton Broth (MHB), and 100 μL of the mixture was dispensed into each well of the Sensititre plate, using the Sensititre AIM. The plate was sealed and incubated at 35 °C in a non-CO_2_ incubator for 7–14 days. After incubation, the results were manually read using the Sensititre Vizion system. Interpretation was made according to the CLSI guidelines (M62, 3rd ed.), for Rapid Growing Mycobacteria (RGM) (Table A1) [19].

### 2.5. Susceptibility Testing to Bedaquiline, Delamanid and Clofazimine by the Broth Microdilution Method

MICs of bedaquiline (TMC207, ref. 20247, Cayman Chemical), delamanid (29697, Cayman Chemicals, Michigan, MI, USA) and clofazimine (1138904, Sigma Aldrich, Milwaukee, WI, USA) were determined using the broth microdilution method, following the Clinical and Laboratory Standards Institute (CLSI) guidelines (M24, 3rd Edition) [18,19]. Bacterial suspensions were inoculated into 96-well microplates containing cation-adjusted Mueller–Hinton broth (CAMHB) supplemented with 5% OADC (oleic acid, albumin, dextrose, and catalase; BD) [20,21]. Serial two-fold dilutions of antibiotics were prepared: 0.06–32 µg/mL for bedaquiline, 0.032–8 µg/mL for delamanid and 0.032–8 for clofazimine, respectively. The control wells included growth control (CAMHB with no antibiotic) and sterility control (no bacterial inoculum). Plates were incubated at 35 ± 2 °C in ambient air and examined after 72 h. MICs were recorded when growth in the control well reached at least a 2+ level (turbidity or visible pellet), as defined by CLSI guidelines [18]. If this threshold was not met, plates were re-incubated and re-read on days 4 and 5, if necessary. If growth remained inadequate on day 5, the test was repeated [18]. Tests were conducted in duplicate in two independent experiments.

### 2.6. Biofilm-Formation Assay by Crystal Violet Staining

Biofilm was analysed by the crystal violet staining method. The bacterial suspensions were inoculated into 96-well plates containing brain–heart infusion broth (BHI) supplemented with 5% OADC and incubated at 37 °C for 7 days. The wells were washed with phosphate-buffered saline (PBS), stained with 0.1% crystal violet for 15 min, and rinsed with distilled water. The bound dye was solubilized with 96% alcohol with acetic acid, and the optical density (OD) was measured at 595 nm (OD_595_). Strains were evaluated as non-biofilm formers (OD of the test isolate  ≤  OD_control_), weak biofilm formers (OD of the test isolate between ODc and 2xOD_control_), moderate biofilm formers (OD of the test isolate between 2 and 4xOD_control_) and strong biofilm formers (OD of the test isolate  >  4xOD_control_) [42]. OD_control_ was calculated as the mean OD of the negative controls plus three standard deviations. All tests were run in duplicate in three independent experiments.

### 2.7. Evaluation of the Antibiofilm Activity of Bedaquiline

To evaluate the impact of bedaquline on biofilm, we evaluated the MBIC value (minimum biofilm inhibitory concentration), defined as the lowest concentration of an antimicrobial agent that prevents visible biofilm formation, assessed by biomass quantification methods such as crystal violet (CV) staining [42]. The MBIC for bedaquiline against 20 *M. abscessus* strains was determined by this method. The experiments were conducted in sterile flat-bottom microtiter plates [42]. Bacterial suspensions were prepared in Middlebrook 7H10 broth supplemented with 5% OADC and with adjusted density of 5 × 10^5^ CFU/mL [18]. Bedaquiline was added to the wells to achieve concentrations ranging from 0.125 to 2 µg/mL Plates were incubated at 37 °C for 7 days. After the incubation period, the wells were gently washed with PBS to remove planktonic cells. The plates were left at room temperature for 24 h until the biofilm was completely dry. The biofilm was stained with 0.1% crystal violet for 15 min and washed with PBS. Then, 96% ethanol with acetic acid was used to dissolve the crystal violet absorbed by the biofilm. The absorbance was measured at 595 nm using a spectrophotometer (Tecan Sunrise; Tecan Group Ltd., Männedorf, Switzerland). The tests were performed in triplicate, with the broth-only wells serving as a negative control.

### 2.8. Bacterial Cell-Viability Assessment and Microscopy

Bacterial cell viability after exposure to the MBIC concentration of bedaquiline was determined using the LIVE/DEAD^®^ BacLight Bacterial Viability Kit (Invitrogen™, Thermo Fisher Scientific, Waltham, MA, USA). The staining procedure was performed according to the manufacturer’s instructions [43]. In brief, dried biofilm samples were stained with 10 µL of a mixture containing diluted fluorescent dyes (SYTO 9 and propidium iodide, PI, in a 1:1 ratio). The samples were incubated in the dark for 15 min and then observed using a Leica Stellaris 8 WLL DLS confocal microscope (Leica Microsystems GmbH, Wetzlar, Germany). Each scan was performed using the same microscope settings, such as resolution (2048 × 2048 pixels), power of the laser and detector, laser frequency (200 Hz) and pinhole (1.0 AU). The samples were scanned with 20×, NA 0.4 objective. The obtained images were analysed in LAS X software (version 4.5) (Leica, Wetzlar, Germany) to evaluate the mean fluorescence intensity. Results are expressed as the ratio of green-to-red fluorescence intensity (live-to-dead cells), according to the cell viability assay used in the study. The scale bar in the images was set to 200 µm.

### 2.9. Statistical Analysis

Associations between colony morphology and biofilm-formation ability were analysed using Fisher’s exact test. A *p*-value of <0.05 was considered statistically significant. The influence of biofilm-forming strength on the bedaquiline MIC values was evaluated using the Mann–Whitney U test. A *p*-value of <0.05 was considered statistically significant. Statistical analysis was performed using Statistica software version 13.0 (StatSoft Inc., Tulsa, OK, USA).

## 3. Results

### 3.1. Molecular Characterisation of Tested M. abscessus Isolates

Molecular analysis of 20 *Mycobacterium abscessus* isolates identified the subspecies distribution as follows: *M. abscessus* subsp. *abscessus* (*n* = 12), *M. abscessus* subsp. *massiliense* (*n* = 7), and *M. abscessus* subsp. *bolletii* (*n* = 1). Subspecies differentiation and resistance mechanism detection for aminoglycosides and macrolides were achieved using the GenoType NTM-DR ver. 1.0 system (Hain Lifescience, Nehren, Germany) (Table 1).

In the analysis of the 13 *M. abscessus* subsp. *abscessus* strains, the results revealed varied responses to macrolides (Table 1) and aminoglycosides. Twelve strains carried the *erm*(41) mutation involving a transition at position T28 (cytosine-to-thymine substitution), which led to macrolide resistance. One strain (no. 9) exhibited additional mutations, including *erm*(41) T28 and transversion in *rrl* gene (A2058C), both conferring resistance to macrolides. In addition, this strain displayed the transversion in *rrs* gene (T1408G), associated with aminoglycoside resistance. One *M. abscessus* subsp. *abscessus* strain (no.1) lacked detectable resistance mutations, suggesting susceptibility to macrolides and aminoglycosides.

Seven strains confirmed as *M. abscessus* subsp. *massiliense* revealed no mutations in the *rrl* and *rrs* genes. Due to a deletion in the *erm*(41) gene in this subspecies, the gene is non-functional, which confers macrolide sensitivity to these strains, regardless of the presence of the *erm*(41) T28 (Table 1).

One strain classified as *M. abscessus* subsp. *bolletii* carried the transition mutation at position T28 of the *erm*(41) gene, which leads to macrolide resistance (Table 1).

These findings underscore the complexity of antibiotic resistance in *M. abscessus* and highlight the necessity for comprehensive genetic analysis to inform effective treatment strategies. The observed variability in macrolide resistance, primarily associated with mutations in the *erm*(41) and *rrl* genes, emphasises the critical importance of individualised antimicrobial therapy based on molecular diagnostics.

### 3.2. Susceptibility of M. abscessus to Therapeutics Routinely Used in RGM Treatment

The MIC values for each antibiotic were evaluated by RAPMYCO2 Sensititre plates (Thermo Fisher, Cleveland, OH, USA). The susceptibility profiles of the tested drugs revealed varying levels of resistance among the strains of the *M. abscessus* complex.

**Figure 1:**
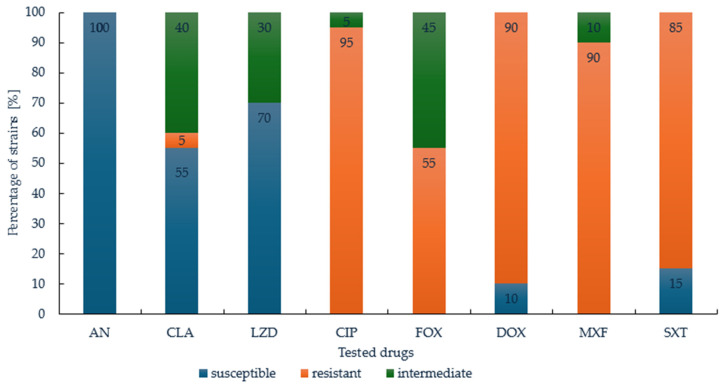
Susceptibility of tested *M. abscessus* complex strains to amikacin (AN), clarithromycin (CLA), linezolid (LZD), ciprofloxacin (CIP), cefoxitin (FOX), doxycycline (DOX), moxifloxacin (MXF) and trimethoprim/sulfamethoxazole (STX). Breakpoint criteria are available in Appendix Table A1.

In total, 100% of the strains were susceptible to amikacin, with MIC values ranging from 1 to 16 µg/mL, underlining its consistent efficacy and making it one of the most effective options in this study. For clarithromycin, 55% of the strains were susceptible, 40% exhibited intermediate susceptibility, and resistance was noted in 5% of the isolates. Linezolid showed promising activity, with 70% of the strains being susceptible, although intermediate susceptibility was present in 30% of cases. Ciprofloxacin showed high levels of resistance, with 95% of strains being resistant and 5% showing intermediate susceptibility (MIC values ranging from 4 µg/mL to 16 µg/mL). Doxycycline also showed limited efficacy, with 90% of the strains being resistant and only 10% susceptible. Among the tested drugs, tigecycline was evaluated, with MIC values ranging from 0.25 to 2 µg/mL However, the strains were not classified as susceptible or resistant, as the clinical breakpoints for tigecycline have not been defined (Figure 1, Table 2).

### 3.3. Potential of Bedaquiline, Delamanid, and Clofazimine Against M. abscessus

Simultaneously with standard drug-susceptibility testing, the microdilution method was conducted to evaluate the activity of bedaquiline, delamanid, and clofazimine against *M. abscessus* strains. According to the obtained results, low MIC values for bedaquiline were observed. Both MIC_50_ and MIC_90_ were determined to be 0.5 µg/mL, and the MIC range extended from 0.125 to 2 µg/mL.

In contrast, delamanid and clofazimine showed limited activity against the strains tested. Delamanid showed MIC values exceeding 16 µg/mL for all isolates, indicating a lack of efficacy in the concentration range tested. Similarly, clofazimine showed MIC values greater than 8 µg/mL for all isolates, suggesting minimal activity against *M. abscessus* (Table 3). Although breakpoints were not yet determined by EUCAST, the obtained results suggest limited clinical utility in treatment regimens.

### 3.4. Differentiation of Morphotypes and Biofilm Formation

**Figure 2:**
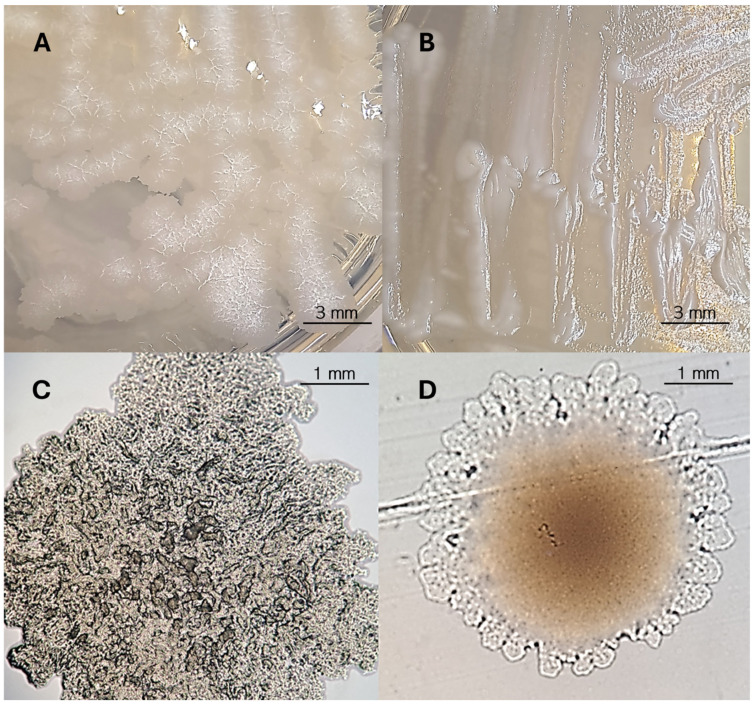
Analysis of *M. abscessus* complex colony morphology on Middlebrook 7H10 agar. (**A**) *M. abscessus* subsp. *abscessus* rough morphotype: irregular, matte colonies with cording. (**B**) *M. abscessus* subsp. *bolletii* smooth morphotype: glossy, translucent colonies with a uniform texture. (**C**) Close-up microscopic view (100× magnification) of a single rough morphotype colony, showing characteristic cording structures. (**D**) Close-up microscopic view (100× magnification) of a single smooth morphotype colony, displaying a uniform, non-cording appearance.

Among the 20 MABc isolates, 16 isolates exhibited a clearly identifiable morphology; 2 isolates (10%) displayed a smooth (S) phenotype, 15 isolates (75%) exhibited a rough (R) phenotype, and 4 (20%) showed mixed morphology (S-R). The smooth morphotype produces glossy, uniform colonies that appear moist and translucent under standard laboratory culture conditions. In contrast, the rough morphotype forms colonies that are irregular, rough-edged, matte, and distinctly waxy in appearance (Figure 2).

*M. abscessus* biofilm-forming strains exhibit a smooth morphotype, forming colonies that are moist, glossy, and translucent. This appearance is attributed to the high expression of glycopeptidolipids (GPLs) on their cell surface, which confer hydrophilic properties. In contrast, rough morphotypes, which have lower levels of (GPLs), present as irregular, rugged colonies, as reported previously [1,42].

Based on the crystal violet (CV) staining method, seven strains were classified as strong biofilm producers (OD_595_/OD_595 control_ > 4). These included all mixed morphotype strains and one smooth strain (*M. abscessus* subsp. *bolletii*), with the mixed morphotypes consistently showing the highest OD_595_ values, indicating a strong biofilm-forming ability. Moderate biofilm formation (OD_595_/OD_595 control_, ranging between 2 and 4) was observed in six strains, mainly exhibiting rough or smooth morphotypes, with the majority of these moderate forms belonging to *M. abscessus* subsp. *abscessus*. In contrast, seven strains were classified as weak biofilm producers (OD_595_/OD_595 control_ < 2), with the rough morphotypes being the most common. These weak biofilm forms were primarily *M. abscessus* subsp. *abscessus* and subsp. *massiliense*, indicating minimal biofilm-formation ability. This distribution indicates a strong relationship between morphotype and biofilm-formation ability (Table 4).

**Figure 3:**
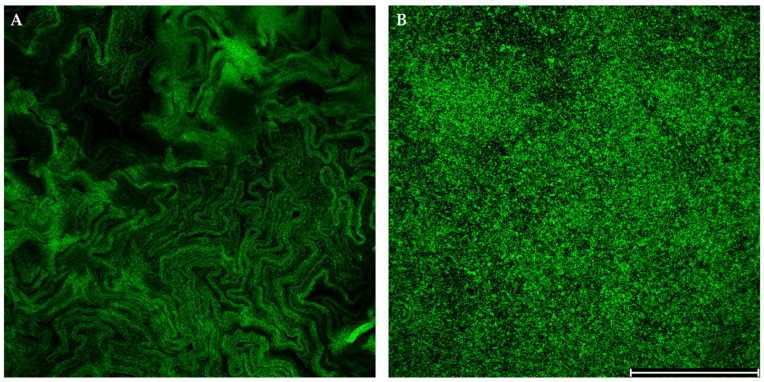
The differences in the cellular arrangement of *M. abscessus* complex strains observed under confocal microscopy using SYTO 9 staining. (**A**) Rough morphotype strain exhibiting dense, cord-like aggregates. (**B**) Smooth morphotype strain displaying a more dispersed, planktonic-like cellular distribution. Scale bar: 200 µm.

The rough morphotype (Figure 3A) typically did not exhibit significant biofilm production. In contrast, the smooth morphotypes of the *M. abscessus* complex which form planktonic-like structures (Figure 3B) were strongly associated with biofilm formation. The mixed morphotype was also a strong biofilm producer. This distinction highlights the potential role of morphotype in determining the pathogenic potential and drug resistance of MABc.

There was a statistically significant association between colony phenotype and biofilm-formation ability among clinical MABc isolates. Strains with a mixed colony morphology were significantly more likely to form strong biofilms (*p* = 0.032, Fisher’s exact test). Although strains with a rough colony morphology tended to form weak biofilms more frequently, this association was not statistically significant (*p* = 0.141, Fisher’s exact test) (Table 5).

### 3.5. Bedaquilline as a Good Antibiofilm Agent

The efficacy of bedaquiline as anti-biofilm agent was demonstrated by MBIC values ranging from 0.25 µg/mL to 2 µg/mL, confirming its effectiveness against *M. abscessus* biofilms. A comparison of MIC and MBIC values revealed that the MBIC/MIC ratio varied between 1 and 4, indicating that higher concentrations of bedaquiline are typically required to inhibit biofilm formation compared to planktonic cells. Interestingly, five strains showed an MBIC/MIC ratio of 1, suggesting that biofilm inhibition in these cases required concentrations similar to those needed for planktonic cell inhibition. However, the most common MBIC/MIC ratio was 4, observed in half of the tested strains, indicating a four-fold increase in the concentration to effectively suppress biofilm growth (Table 6).

**Figure 4:**
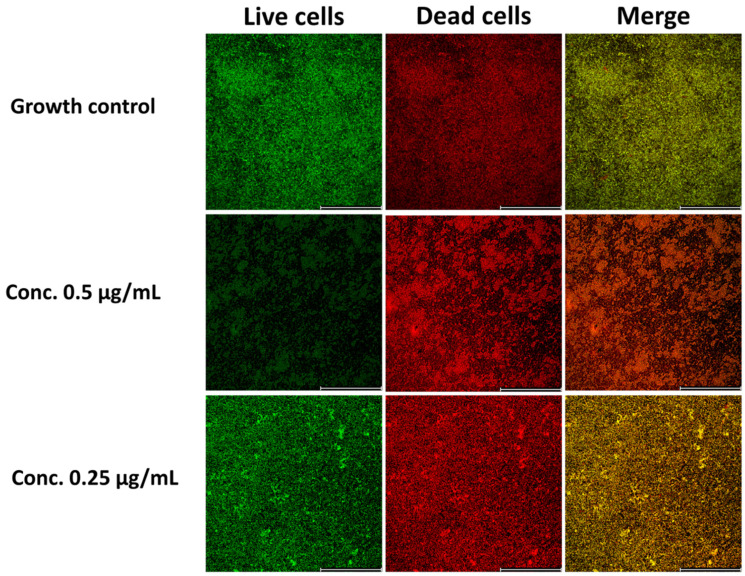
Confocal microscopy images of *M. abscessus* biofilm stained with LIVE/DEAD^®^ assay and treated with different concentrations of bedaquiline. The panels show the growth control (untreated biofilm) and biofilms treated with 0.5 µg/mL and 0.25 µg/mL, illustrating live cells (green), dead cells (red), and merged fluorescence channels. Scale bar: 200 µm.

Additionally, the analysis of bacterial cell viability in biofilm structure was conducted using the LIVE/DEAD^®^ bacterial viability assay with confocal microscopy visualization. The results revealed that bedaquiline significantly inhibited biofilm formation at the tested concentrations. At 0.5 µg/mL, corresponding to MIC values, the reduction in biofilm formation was 45.24% compared to the growth control, while at 0.25 µg/mL and 0.125 µg/mL, the reductions were 29.76% and 13.10%, respectively (Figure 4). These findings demonstrate that bedaquiline significantly inhibits biofilm formation at concentrations corresponding to its MIC, but does not fully eradicate biofilms. This supports its potential as a valuable agent for limiting biofilm formation by *M. abscessus*.

Additionally, we found that there was no statistically significant difference in bedaquiline MIC values between weak and moderate/strong biofilm-forming *M. abscessus* complex isolates (*p* = 0.828). The mean MIC was 0.438 µg/mL for the weak biofilm formers and 0.425 µg/mL for the moderate/strong biofilm formers.

## 4. Discussion

Treating *M. abscessus* complex infections is highly challenging, due to the bacterial resistance mechanisms and its ability to form biofilms. Furthermore, the accurate identification and differentiation of MABc subspecies, including *M. abscessus* subsp. *abscessus*, *massiliense*, and *bolletii*, remain critical because these subspecies exhibit significant differences in virulence, biofilm-formation capacity, and susceptibility to antimicrobials. Rapid genetic tests, such as molecular tests targeting resistance genes or subspecies-specific markers, provide valuable tools to guide treatment decisions and optimise antimicrobial therapy.

The analysis of 20 *M. abscessus* isolates revealed a diverse subspecies distribution, with *M. abscessus* subsp. *abscessus* being the most prevalent (*n* = 12), followed by *M. abscessus* subsp. *massiliense* (*n* = 7) and *M. abscessus* subsp. *bolletii* (*n* = 1). The identification of subspecies is clinically significant because of their differing resistance profiles and therapeutic implications [5,7]. A high prevalence of macrolide resistance was observed in 70% (14/20) of the isolates, consistent with the frequent occurrence of mutations in the *erm*(41) or *rrl* genes (in the one isolate). The *erm*(41) T28C mutation, which confers inducible macrolide resistance, was particularly prominent in subsp. *abscessus*. These findings underline the significant challenge macrolide resistance poses for effective treatment, especially given the reliance on macrolides as a cornerstone of MABc therapy [16,44,45,46]. *M. abscessus* subsp. *abscessus* is often associated with poor treatment outcomes due to inducible macrolide resistance, mediated by a functional *erm*(41) gene. In contrast. *M. abscessus* subsp. *massiliense* typically lacks this functional gene, resulting in higher macrolide susceptibility and better clinical responses [17,47]. Multiple studies have examined the roles of *erm*(41) and *rrl* mutations in *M. abscessus* resistance to clarithromycin [48,49], although their findings have been inconsistent. These mutations do not account for all clarithromycin-resistant *M. abscessus* strains [50], suggesting the presence of additional resistance mechanisms [44,50,51,52,53]. In contrast, aminoglycoside resistance was rare, detected in only 5% (1/20) of the isolates. This finding aligns with the established efficacy of aminoglycosides, such as amikacin, against MABc. The low rate of resistance supports the continued use of amikacin as a key therapeutic agent in combination regimens, while also underscoring the importance of routine surveillance to monitor potential resistance development.

The study also highlighted widespread resistance to ciprofloxacin, moxifloxacin, and doxycycline, limiting the effectiveness of these agents for MABc treatment. The susceptibility results for linezolid and trimethoprim–sulfamethoxazole were variable, with some isolates showing intermediate or resistant profiles. This variability underscores the importance of cautious clinical use and emphasises the need for tailored treatment plans based on individual susceptibility results.

Exploring new treatment options for *M. abscessus* infections among drugs already approved for *M. tuberculosis* therapy appears to be a promising alternative. Therefore, we evaluated the activity of bedaquiline, delamanid, and clofazimine against 20 clinical *M. abscessus* strains. Our results revealed low MIC values for bedaquiline, with MIC_50_ and MIC_90_ both determined to be 0.5 µg/mL and an MIC range of 0.125 to 2 µg/mL. The findings of this study underscore the significant potential of bedaquiline as an effective therapeutic option for *M. abscessus* infections. Bedaquiline has been approved by the US Food and Drug Administration (FDA) and the European Medicines Agency for the treatment of multidrug-resistant tuberculosis [53]. In vitro, it demonstrates bacteriostatic activity against *M. abscessus* isolates, with an MIC_50_ of 0.125 μg/mL and an MIC_90_ greater than 16 μg/mL [53].

Bedaquiline shows moderate activity based on epidemiological cutoff values, with its efficacy significantly enhanced when used in combination therapies [54]. Moigne et al. [54] demonstrated that the combination of bedaquiline and imipenem significantly enhanced infection clearance, emphasizing the importance of evaluating antibiotic combinations rather than relying solely on monotherapies when addressing highly drug-resistant mycobacteria. Similarly, Ruth et al. [55] evaluated the effectiveness of bedaquiline against *M. abscessus* infections. Their study revealed concentration-dependent bacteriostatic activity, although its efficacy was lower against *M. abscessus* than *M. avium*. A synergistic effect was observed when bedaquiline was combined with clofazimine, whereas no interaction was noted with clarithromycin. Resistance to bedaquiline developed during prolonged exposure, and, in vivo, bedaquiline monotherapy failed to prevent mortality in a murine *M. abscessus* model. These findings indicate that while bedaquiline exhibits bacteriostatic effects, its therapeutic potential against *M. abscessus* is limited, especially when used as monotherapy [25,54,55,56].

Delamanid and clofazimine exhibited limited activity against *Mycobacterium abscessus* in this study, with minimum inhibitory concentration (MIC) values exceeding 16 µg/mL and 8 µg/mL, respectively. These results align with previous reports indicating the poor efficacy of these agents against *M. abscessus* [25,57]. While clofazimine demonstrated effectiveness against other non-tuberculous mycobacteria (NTM), particularly the *M. avium* complex (MAC), its role in *M. abscessus* infections remains uncertain [17,44].

Combination therapies, particularly those involving bedaquiline, clofazimine, delamanid and other antimicrobials, are being increasingly explored. Studies suggest that combining delamanid with other agents may result in synergistic effects, offering a potential avenue for improving treatment efficacy. However, further investigation into optimal drug combinations, dosing regimens, and host-directed therapies is necessary to enhance treatment outcomes [21,25,26,56,58,59].

Beyond antimicrobial resistance, another key challenge in treating *M. abscessus* infections is biofilm formation, which enhances bacterial survival and limits antimicrobial efficacy in hostile environments [60,61]. This process is influenced by multiple factors, with evidence linking colony morphotype to biofilm-forming capacity [62,63].

We analysed the distribution of morphotypes among the tested MABc strains. The majority of the isolates (70%, 14/20) exhibited the rough morphotype, while only 10% (2/20) presented the smooth morphotype, typically linked to lower virulence and increased susceptibility to host immune defences [64]. Statistical analysis revealed a significant association between colony morphology and biofilm-formation capacity, with mixed morphotypes being significantly more likely to form strong biofilms (*p* = 0.032). However, due to the variability observed within each morphotype group, further studies with larger strain collections are necessary to confirm and better understand these associations.

The predominance of the rough morphotype in this study is consistent with previous reports, where this phenotype has been associated with more severe clinical outcomes, higher virulence, persistence in host tissues, and more pronounced inflammatory responses [60,65,66,67]. The underlying factors behind increased pathogenicity are not fully understood; however, rough *M. abscessus* strains exhibit this [46]. Rough variants also display increased cording, a virulence factor in mycobacterial infections that impairs macrophage phagocytosis [46,68]. The presence of both smooth and rough colonies within the same strain may represent a strategy to maximise survival in fluctuating environments, where one morphotype might be more suited for persistence in certain conditions, while the other could offer better survival under different circumstances. This finding aligns with the notion that *M. abscessus* populations are genetically diverse and capable of adapting rapidly to changes in their environment [69,70]. However, studies on the antibiotic susceptibility of *M. abscessus* isolates from both rough and smooth morphotypes have yielded inconsistent findings [60,66,71,72].

The formation of biofilms is a key factor in the pathogenicity of MABc [69]. The biofilm-forming capability of the MABc strains is a critical factor contributing to their persistence and resistance to antimicrobial agents. The success of new therapies will heavily depend on their ability to penetrate and dismantle biofilms, as biofilms are responsible for the majority of chronic and recurrent bacterial infections [73].

In this study, 25% (5/20) of the strains were strong biofilm formers, while 75% (15/20) demonstrated weak-to-moderate biofilm formation. Biofilm-positive strains pose a significant challenge in clinical settings, as biofilm formation increases the tolerance to antibiotics and host immune responses, complicating eradication efforts. The low prevalence of biofilm-positive strains may suggest that strain-specific differences in biofilm-forming capacity or environmental conditions influence this phenotype. Nevertheless, the identification of biofilm-positive strains highlights the need for therapies targeting biofilm-associated resistance mechanisms, such as combination treatments or biofilm-disrupting agents. Biofilm structures are complex, and present key challenges in measuring the number of active cells, biofilm morphology, and mass accumulation. The findings of this study emphasise the complexity of managing MABc infections due to diverse resistance profiles, morphotype variability, and biofilm-formation capabilities. The high prevalence of macrolide resistance necessitates alternative therapeutic strategies, such as the use of second-line agents or combination therapies. Additionally, the predominance of rough morphotypes underscores the aggressive nature of many MABc infections, which may require more intensive management. Future studies should focus on elucidating the molecular mechanisms underlying these phenotypes and their clinical impact. Integrating phenotypic and genotypic data will enhance our understanding of MABc pathogenicity and inform the development of a targeted therapeutic approach.

Bedaquiline’s antibiofilm activity, with MBIC values between 0.25 and 2 µg/mL, further strengthens its case for use as a key antimicrobial for tackling *M. abscessus*. The MBIC/MIC ratio, which was most commonly observed as 4, suggests that while higher concentrations are required to inhibit biofilm growth compared with planktonic cells, these values remain within achievable therapeutic ranges. Confocal microscopy and the LIVE/DEAD^®^ assay revealed a significant reduction in biofilm viability, with bedaquiline reducing biofilm mass by 45% at MBIC concentrations. This ability to suppress biofilm formation, rather than simply eradicating existing biofilms, could play a critical role in preventing chronic biofilm-related complications. It is worth noting that the interpretation of viability staining in *Mycobacterium* spp. may be limited by the lipid-rich, hydrophobic cell wall, which can impair the penetration of fluorescent dyes such as propidium iodide. As previously reported, this may lead to inaccurate differentiation between viable and non-viable cells when using conventional viability kits based on membrane integrity [74].

Our study focused on evaluating the MBIC of bedaquiline, which measures its ability to prevent biofilm formation when administered at the onset of bacterial growth. While MBEC (Minimum Biofilm Eradication Concentration) is commonly used to assess the ability of an agent to eliminate pre-formed biofilms, MBIC is equally important in the context of *M. abscessus* infections. Inhibiting biofilm formation at an early stage can significantly impact bacterial persistence, as biofilms contribute to increased antimicrobial resistance, host immune evasion, and chronic infections. Moreover, MBIC testing provides insights into the effects of bedaquiline on the initial adhesion phase and early biofilm development, which are critical steps in biofilm formation.

The choice of culture medium is a critical factor influencing biofilm formation in *M. abscessus* [75,76]. To date, there is no universally standardized protocol for biofilm assays in RGM, and various media including Middlebrook 7H9, Sauton’s medium, or CAMHB have been employed across studies [76]. In the present study, BHI broth supplemented with 5% OADC was selected for the biofilm-formation assay. Although BHI is not the most commonly used medium for *M. abscessus*, it is widely applied in crystal violet-based biofilm quantification protocols for other bacteria [77,78], and this approach was extrapolated to *M. abscessus* in our experimental setup. The potential influence of medium composition on the characteristics and biomass of biofilms observed in this study should be considered when interpreting the results.

The findings of our study underscore the significant potential of bedaquiline as an effective therapeutic option for *M. abscessus* infections. This is particularly noteworthy, given the resistance mechanisms of *M. abscessus* and the role of biofilms in promoting persistence and the treatment failure.

In recent years, there has been a growing focus on *M. abscessus* biofilm research. However, the absence of standardised methods for culturing, characterising, and testing drug activity in biofilms makes it difficult to compare results across different studies. Variations in the biofilm models and experimental conditions used in *M. abscessus* biofilm research can influence the biofilm’s properties and behaviour, which helps explain the wide range of seemingly contradictory findings in this area of study. This study emphasizes the need for personalized treatment strategies and underscores the importance of frequent and thorough susceptibility testing in managing *M. abscessus* infections. This strategy will be crucial in addressing and overcoming the challenges posed by antimicrobial resistance in these highly resistant and difficult-to-manage bacterial infections.

## 5. Conclusions

To sum up, the results of our study highlight the significant therapeutic potential of bedaquiline against the *M. abscessus* complex, particularly in biofilm-forming strains, with consistent MIC_50_ and MIC_90_ values of 0.5 µg/mL. Importantly, despite the well-known challenge of biofilm-associated resistance, bedaquiline maintained its activity irrespective of the biofilm-forming capacity, with no statistically significant differences observed between weak and moderate/strong biofilm producers (*p* = 0.828). Furthermore, the MBIC values of bedaquiline were only up to four-fold higher than the MIC values, confirming effective biofilm penetration. Delamanid and clofazimine showed limited activity, with high MIC values exceeding 16 µg/mL and 8 µg/mL, respectively. The observation that 25% of the strains formed strong biofilms underscores the clinical importance of targeting biofilm structures, which contribute to persistence and therapeutic failure. These findings strongly support the inclusion of bedaquiline as a core component of personalised, combination regimens to overcome the dual challenges of biofilm formation and multidrug resistance in *M. abscessus* complex infections.

## Figures and Tables

**Table 1:** Characteristics of *M. abscessus* complex strains—profiles of resistance to macrolides and aminoglycosides obtained by both phenotypic method and by molecular methods.

No.	Species	Macrolide Resistance			Aminoglycoside Resistance		
		Clarithromycin MIC (µg/mL)		Detected Mutation	Amikacin MIC (µg/mL)		Detected Mutation
1	*M. abscessus* subsp. *abscessus*	0.12	S	-	16	S	-
2	*M. abscessus* subsp. *abscessus*	8	R	*erm*(41) T28	16	S	*-*
3	*M. abscessus* subsp. *abscessus*	8	R	*erm*(41) T28	16	S	*-*
4	*M. abscessus* subsp. *abscessus*	16	R	*erm*(41) T28	16	S	*-*
5	*M. abscessus* subsp. *abscessus*	16	R	*erm*(41) T28	16	S	*-*
6	*M. abscessus* subsp. *abscessus*	8	R	*erm*(41) T28	16	S	*-*
7	*M. abscessus* subsp. *abscessus*	16	R	*erm*(41) T28	16	S	*-*
8	*M. abscessus* subs *massiliense*	0.125	S	-	16	S	-
9	*M. abscessus* subsp. *abscessus*	16	R	*erm*(41) T28, *rrl*	16	S	*rrs*—T1408G
10	*M. abscessus* subs *massiliense*	0.06	S	-	4	S	-
11	*M. abscessus* subs *massiliense*	0.06	S	-	16	S	-
12	*M. abscessus* subsp. *bolletii*	8	R	*erm*(41) T28	16	S	*-*
13	*M. abscessus* subsp. *abscessus*	0.25	S	*erm*(41) T28	16	S	*-*
14	*M. abscessus* subsp. *abscessus*	16	R	*erm*(41) T28	16	S	*-*
15	*M. abscessus* subsp. *abscessus*	0.5	S	*erm*(41) T28	2	S	*-*
16	*M. abscessus* subs *massiliense*	0.12	S	-	4	S	-
17	*M. abscessus* subs *massiliense*	≤0.06	S	-	≤1	S	-
18	*M. abscessus* subs *abscessus*	0.06	S	*erm*(41) T28	16	S	*-*
19	*M. abscessus* subsp. *massiliense*	0.06	S	-	16	S	-
20	*M. abscessus* subsp. *abscessus*	0.5	S	*erm*(41) T28	2	S	*-*

R—resistant, S—susceptible.

**Table 2:** Distributions of MIC_50_ and MIC_90_ values for 20 isolates of *M. abscessus* complex in the RAPMYCO panel.

Antimicrobial Agent	MIC (μg/mL)		
	MIC_50_	MIC_90_	Range
AN	16	16	1–16
FOX	128	128	32–128
CIP	4	4	2–4
DOX	16	16	1–16
LZD	2	16	1–16
MXF	4	8	2–8
SXT	4/76	8/152	4/76–8/152
TGC	0.5	1	0.25–2
CLA	0.06	4	0.5–16

AN—amikacin, FOX—cefoxitin, CIP—ciprofloxacin, DOX—doxycycline, LZD—linezolid, MXF—moxifloxacin, SXT—trimethoprim/sulfamethoxazole, TGC—tigecycline, CLA—clarithromycin. MIC_50_ refers to the minimum inhibitory concentration at which 50% of the tested bacterial isolates are inhibited. MIC_90_ is the concentration at which 90% of the isolates are inhibited.

**Table 3:** Distributions of MIC_50_ and MIC_90_ values for 20 isolates of *M. abscessus*.

	MIC (μg/mL)		
Antimicrobial Agent	MIC_50_	MIC_90_	Range
BDQ	0.5	0.5	0.125–1
DEL	>16	>16	>16
CLO	>8	>8	>8

BDQ—bedaquiline, DEL—delamanid, CLO—clofazimine. MIC_50_ refers to the minimum inhibitory concentration at which 50% of the tested bacterial isolates are inhibited. MIC_90_ is the concentration at which 90% of the isolates are inhibited.

**Table 4:** The analysis of *M. abscessus* morphotypes on Middlebrook 7H10 Agar and biofilm formation by crystal violet staining.

Strains No.	Species	Morphotype 7H10 Agar	Crystal Violet Staining Method		
			OD_595_	OD_595_/OD_595control_ Ratio *	Characterization
1	*M. abscessus* subsp. *abscessus*	Rough	2.45	1.99	weak biofilm
2	*M. abscessus* subsp. *abscessus*	Rough	4.89	3.74	moderate biofilm
3	*M. abscessus* subsp. *abscessus*	Rough	2.35	1.76	weak biofilm
4	*M. abscessus* subsp. *abscessus*	Smooth	4.69	3.59	moderate biofilm
5	*M. abscessus* subsp. *abscessus*	Rough	3.78	3.15	moderate biofilm
6	*M. abscessus* subsp. *abscessus*	Mixed	5.98	4.63	strong biofilm
7	*M. abscessus* subsp. *abscessus*	Mixed	5.98	4.96	strong biofilm
8	*M. abscessus* subs *massiliense*	Rough	2.08	1.64	weak biofilm
9	*M. abscessus* subsp. *abscessus*	Rough	2.39	1.98	weak biofilm
10	*M. abscessus* subsp. *massiliense*	Rough	1.27	1.02	weak biofilm
11	*M. abscessus* subsp. *massiliense*	Rough	3.56	3.02	moderate biofilm
12	*M. abscessus* subsp. *bolletii*	Smooth	5.43	4.55	strong biofilm
13	*M. abscessus* subsp. *abscessus*	Rough	4.86	3.51	moderate biofilm
14	*M. abscessus* subsp. *abscessus*	Rough	2.81	1.90	weak biofilm
15	*M. abscessus* subsp. *abscessus*	Rough	2.25	1.73	weak biofilm
16	*M. abscessus* subsp. *massiliense*	Rough	5.48	4.34	strong biofilm
17	*M. abscessus* subsp. *massiliense*	Mixed	1.93	1.55	weak biofilm
18	*M. abscessus* subs *abscessus*	Rough	2.23	1.44	weak biofilm
19	*M. abscessus* subsp. *massiliense*	Rough	2.54	1.37	weak biofilm
20	*M. abscessus* subsp. *abscessus*	Mixed	5.98	4.76	strong biofilm

* OD_595_/OD_595_NC Ratio—strains were evaluated as non-biofilm producers (OD of the test isolate  ≤  OD_control_), weak biofilm producers (OD of the test isolate between ODc and 2xOD_control_), moderate biofilm producers (OD of the test isolate between 2 ODc and 4xOD_control_) and strong biofilm producers (OD of the test isolate  >  4xODc). 7H10—Middlebrook agar.

**Table 5:** Association between colony morphotype and biofilm-formation ability in clinical MABc isolates.

Phenotype of Colony	Ability to Form Biofilm	No. of Strains	Comparison and *p*-Value *
Rough	weak biofilm	9	Rough vs. Weak: *p* = 0.141 (ns ^a^)
	moderate biofilm	4	
	strong biofilm	1	
Smooth	moderate biofilm	1	Smooth vs. Strong: *p* = 0.447 (ns ^a^)
	strong biofilm	1	
Mixed	weak biofilm	1	Mixed vs. Strong: *p* = 0.032 (s ^b^)
	strong biofilm	3	

* Statistical significance assessed by Fisher’s Exact Test; *p* < 0.05 considered significant. ^a^ ns—non-significant, ^b^ s—significant.

**Table 6:** Comparison of bedaquiline activity against planktonic cells and biofilm defined by MIC and MBIC values, respectively.

Tested Strains	Bedaquiline Activity		
	MIC (µg/mL)	MBIC (µg/mL)	MBIC/MIC Ratio
1	0.5	2	4
2	0.5	1	2
3	1	1	1
4	0.5	1	2
5	0.5	0.5	1
6	0.5	1	2
7	0.5	2	4
8	0.5	1	2
9	0.25	1	4
10	0.25	1	4
11	0.5	2	4
12	0.25	1	4
13	0.5	1	2
14	0.25	1	4
15	0.5	1	2
16	0.25	1	4
17	0.125	0.25	2
18	0.5	2	4
19	0.5	2	4
20	0.25	1	4

## Data Availability

The original contributions presented in this study are included in the article. Further inquiries can be directed to the corresponding author.

## Abbreviations

The following abbreviations are used in this manuscript:

BDQ	Bedaquiline
CFU	Colony-Forming Units
CLO	Clofazimine
DEL	Delamanid
CV	Crystal Violet
LJ	Löwenstein–Jensen medium
MABc	*Mycobacterium abscessus* complex
MBIC	Minimal Biofilm Inhibitory Concentration
MIC	Minimal Inhibitory Concentration
NTM	Non-Tuberculous Mycobacteria
OD	Optical Density
PI	Propidium Iodide
DST	Drug Susceptibility Testing

## Appendix A

**Table A1:** Antimycobacterial agents and breakpoints for testing rapidly growing Mycobacteria [19].

Antimicrobial Agent	MIC (µg/mL)		
	S	I	R
Amikacin (IV)	≤16	32	≥64
Cefoxitin	≤16	32–64	≥128
Ciprofloxacin	≤1	2	≥4
Clarithromycin	≤2	4	≥8
Imipenem	≤4	8–16	≥32
Linezolid	≤8	16	≥32
Meropenem	≤4	8–16	≥32
Moxifloxacin	≤1	2	≥4
Trimetoprim-sulfametoxazol	≤2/38	-	≥4/76
Tigecycline	-	-	-
Tobramycin	≤2	4	≥8
